# A Systematic Review of Technology-Enhanced Project-Based Language Learning: Theoretical Frameworks, Project Types, and Implications for Future Research and Practice

**DOI:** 10.12688/f1000research.165491.1

**Published:** 2025-08-08

**Authors:** Ainun Fikria, Slamet Setiawan, Ahmad Munir

**Affiliations:** 1 Language and Literature Education, Universitas Negeri Surabaya, Surabaya, East Java, Indonesia

**Keywords:** Project-based language learning, educational technology, computer assisted language learning, technology enhanced language learning, systematic literature review.

## Abstract

**Background:**

This paper aims to revisit the nature of published research on technology-enhanced project-based language learning (TEPBLL), exploring the conceptual framework or theories pertaining to TEPBLL, type of projects, and language knowledge or skills that could be promoted by TEPBLL, and showcases the implications derived from the application of TEPBLL.

**Methods:**

Limited to high-quality SSCI publications and four gates of exclusion criteria, this research examined 31 articles on technology-enhanced project-based language learning applying PRISMA 2020 protocol, focusing on five aspects: (1) the nature of the publications, (2) the conceptual frameworks used in previous research, (3) the types of projects used in TEPBLL and its effectiveness in students’ language skills and knowledge, and (4) the implications of the studies.

**Results:**

The findings revealed five theoretical frameworks, seven types of projects in TEPBLL, and eight major skills and knowledge that were promoted by technology-enhanced project-based language learning, and showcased three dimensions of implications for future research and practice in language education.

**Conclusions:**

This study thoroughly examined TEPBLL on students’ language skills, language knowledge, and affective status through a systematic literature review. Furthermore, this study provided valuable insights for teachers, language facilitators, and administrators to consider the potential of TEPBLL in their classrooms.

## Introduction

Technology-enhanced learning has been a persistent topic in education as it engages students ( Munaro et al., 2021) and encourages teachers ( Garib, 2023). As the demeanors of technology reduce meaningful learning, project-based language learning looks at the possibility and learning benefits. Technology continues to play a crucial role in modern approaches towards education, offering opportunities to improve the language learning process. As rooted in the constructivist approach, Project-based learning (PBL) is a type of learning that focuses on meaningful and student-centered project-based activities in carrying out projects.

The use of technology within PBL contexts could enhance learner engagement and collaboration, while offering opportunities for relevant and practical language use. Much research has been conducted on the use of technology in language acquisition and language teaching. Therefore, a meta-analysis of the technology types and their usefulness for the FLT process and an analysis of the effect of specific technologies on the development of foreign language proficiency should be conducted ( Golonka et al., 2012). In the literature review section, the author discussed the application of technology in the teaching and learning of the English language, especially in the context of technological advancement in the teaching of skills in the English language ( Ahmadi, 2018). PBL also offered an understanding of how technology-aided language learning focuses on how to use technology to enrich and facilitate language learning ( Zhou & Wei, 2018).

As language learning becomes less meaningful without concern for content learning, project-based language learning (PBLL) is gaining more attention because it enables the educators to develop engaging learning environments that enhance students’ creative thinking, innovation, problem-solving abilities, and communication skills ( Sun & Yang, 2013; Syzenko & Diachkova, 2020). The use of technology in language teaching has been highlighted as a dynamic process because of the constant interaction between technology and the practical application of educational principles in helping language learning ( Palanisamy, 2024). However, studies of chatbot-based language learning support, adaptive learning algorithms, and virtual reality language immersion have tried to add value to the current literature on technology-enhanced language learning and have practical implications for educators and practitioners. Bahari (2024) highlighted the potential benefits of using technology in language learning including its meta-analysis results on virtual reality–assisted language learning, which mentioned that VR has the potential to engage students in real-life situations.

When adopting technology in project-based language learning, which is referred to as TEPBLL, researchers established that learner shifted from being mere recipients of information to active consumers of knowledge, enriched student-centered, learner-directed, project-based activities, and promoted creativity among learners ( Fan, 2024; Lin & Wang, 2023; H. C. Yeh & Fu, 2023). Thus, for a better understanding of TEPBLL, the present study revisited all the publications in the field of TEPBLL’s dimensions within a decade (2015-2024) using PRISMA 2020 protocol. This paper aims to explain the nature of published research on TEPBLL, exploring the framework theories covering TEPBLL, the type of technologies and language knowledge or skills that could be promoted by TEPBLL, and implications derived from the application of TEPBLL.

### The concept of technology-enhanced project based language learning

Technology-enhanced project-based language learning (TEPBLL) is an innovative approach to education that incorporates the use of projects in learning a second language and technology in the authentic use of the second and/or foreign language. This approach involves the use of modern technology tools and platforms to complete project-based assignments that involve the use of oral and written language, innovatively created to enable technical, collaborative, and real-life assignments ( Dooly et al., 2021; Syzenko & Diachkova, 2020). TEPBLL consequently delineates the possibilities for student engagement in 21st-century knowledge, skills, and competencies in project-based language learning through technology-enhanced tasks and activities that enhance learning experiences ( Dooly et al., 2021). In PBLL, the project’s objective is authentic and the target audience for its result is real because it is relevant to circumstances outside of the classroom.

This enables educators to develop engaging learning environments that enhance students’ creative thinking, innovation, problem-solving abilities, and communication skills ( Sun & Yang, 2013; Syzenko & Diachkova, 2020). As a theory, TEPBLL holds that learners are more likely to acquire a second language when such learning is a by-product of the need to use a target language for meaningful tasks and purposes. Project-based language learning via technology, namely TEPBLL, allows for the effective use of technology in facilitating language acquisition which can enable students to achieve efficient verbal communication in real-life situations, as supported by Nur et al. (2022) and Syzenko & Diachkova (2020). Overall, TEPBLL is a teaching strategy that integrates technology into project-based learning environments, aimed at effectively and meaningfully improving students’ language abilities, critical thinking skills and digital literacy.

## Methods

This study adhered to a three-stage approach of literature research, article screening, and data analysis referred to PRISMA 2020 checklist ( Fikria et al., 2025b). For the article search, we utilized Web of Science (WoS) and limited our studies to SSCI journals only, like some of the prior reviews (e.g. Su & Zou, 2020; Zou et al., 2019). This was because the majority of editorial papers, book chapters, conference papers, and non-SSCI journal articles we reviewed had little of information that could be helpful for our review and analysis, such as the theoretical frameworks and technology used in language knowledge and skill acquisition. We accustom some of these publications in the initial stage of the study but we felt that they are not suitable for detailed analysis; several had no theoretical framework, while other explored the technology in isolation rather than focusing on its application in second language acquisition. Thus, the decision was made to exclude articles that did not belong to SSCI as the overall journals were of relatively high quality, and the articles contained all the necessary information for the analysis of the review. The same research techniques were employed in studies by Zhang and Zou (2020) and Zou et al. (2019).

### Inclusion and exclusion convention

To find relevant articles, we used the Web of Science with the following Boolean search syntax, “project-based language learning” and ‘technology’ or ‘project’ and ‘English language learning’. We used the period of interest as 2015-2024 and chose the literature source as the SSCI and found 319 articles. Four gates of exclusion criteria were initially identified. We excluded article that were: a review articles, editorials, and viewpoints; not focus on project-based language learning; not employing technology; using technology for scoring. We included articles that: were empirical studies, presented the technology integrated into project-based language learning, provided clear description of the technology used.

### Coding process

Three of the researchers, employed the bottom-up approach and analyzed 31 articles using the five research questions developed for the study. I found that there were differences in the nature of publications based on the year of publication, the journal in which they were published, and whether the authors were affiliated to a university or not. The theories, techno collaborations that improved the technologies, applicability of TEPBLL, and implications were categorized as outlined in the articles. First, the relevant contents extracted from the reviewed articles were described in words; all of the following steps were taken literally: sorting all the results by similarity, which are made in order to create dataset tables ( Fikria et al., 2025a) that help to summarize results. This coding strategy from the bottom up helped the researchers to reduce the interpretational biases during the study and provided a correct picture of the research studies that were reviewed. For example, to code the conceptual frameworks or the theory involved in the 31 studies the researchers took the following steps: the researchers started by reading one article at a time very carefully, noted down the conceptual frameworks or theories mentioned in the papers, combined the same concepts together, and finally prepared a list of the main conceptual frameworks and theories.

Two researchers mutually coded three articles selected at random and then discussed and compared codes and came down to fine-tune the articles until both readers had a similar understanding of the selected articles as well as the coding frame work before they were allowed to self-code the rest of the selected articles. In this process, both the researchers always remembered that which we are going to code should be the actual meanings of the articles and then they read the articles again where they simply summarized what those articles described in terms of the theoretical frameworks, technologies, research findings and implications and then only coded them. Subsequently, the labels were grouped and categorized into groups of similarity and subdivided as necessary with extra caution. For example, create VR video using presentation, practice, and production procedure ( Shi et al., 2024), students determine their own project, collected data, and discussed the data ( Hsieh et al., 2022), students also synthesized data and conclusions ( Tanaka, 2023) were coded as a ‘student activity’. If there was any disagreement regarding some aspect of the data, an analysis of the results was conducted by discussion with the third researcher.

## Results

**Figure 1.**
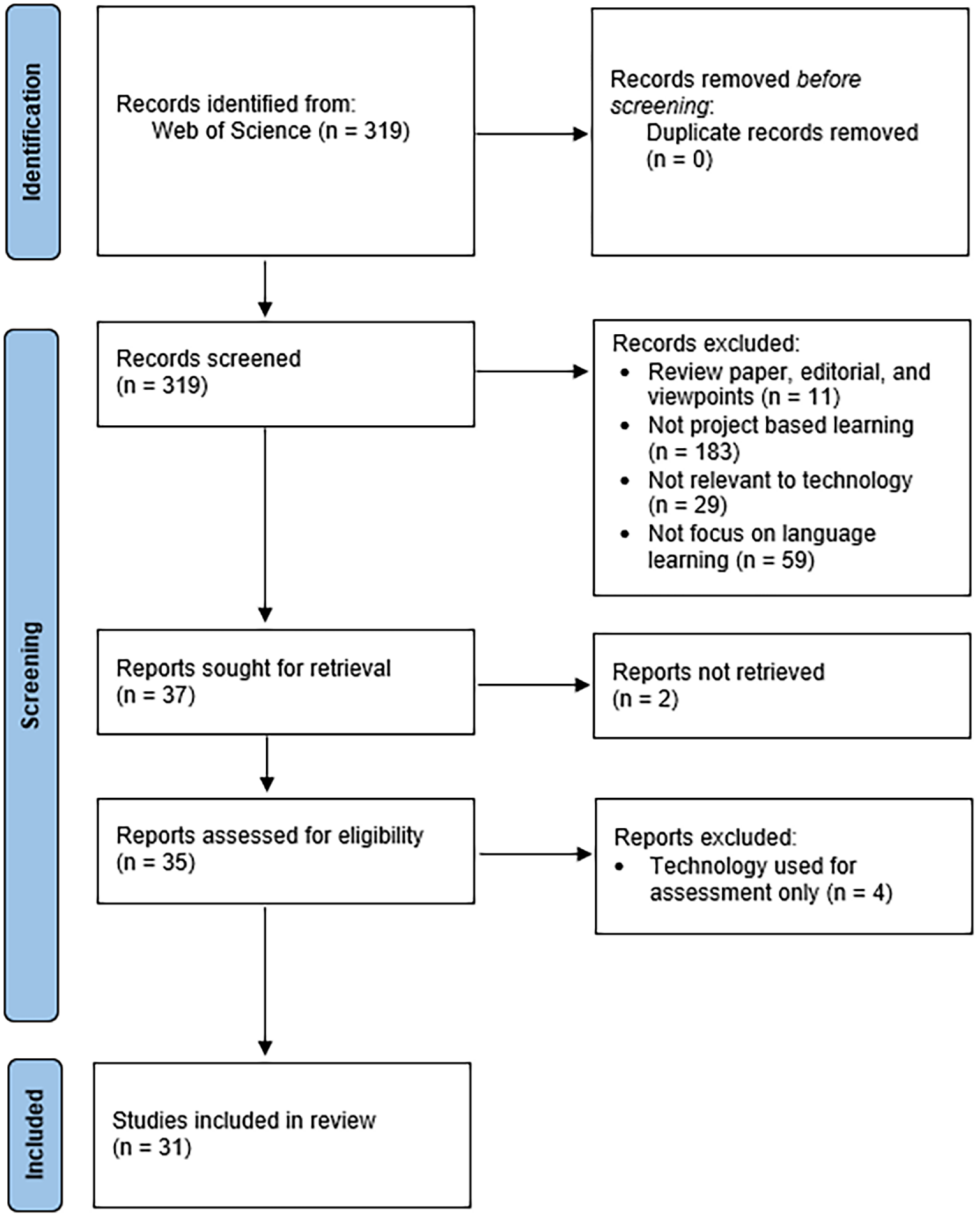
PRISMA 2020 flow diagram.

The findings section consists of the nature of published research on TEPBLL, exploring the framework theories covering TEPBLL, the type of projects used in TEPBLL, type of language knowledge and skills that could be promoted by TEPBLL, implications derived upon the application of TEPBLL, and discussion on the steps involved in conducting TEPBLL in all contexts. The flow chart in Figure 1 portrays the research and selection of eligible studies. The initial research generated total of 319 articles. After checking duplication, it remains 319 articles to be identified for title and abstract screening. 37 full-text were included for further analysis. 31 of them met the eligibility criteria that were included in the study.

### The nature of published research on TEPBLL

**Figure 2.**
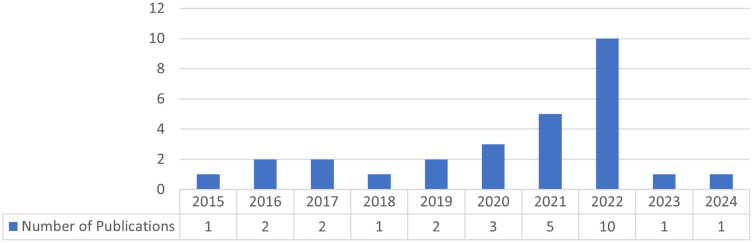
Publication date.

Research in technology enhanced project-based language learning has been gaining a stable trend in recent decades, and an increasing trend in 2022 ( Figure 2).

After analyzing the 31 articles that met the criteria, the result was described as follows: a total of 31 articles were published in 21 SSCI journals, reflecting the field’s overall publication situation ( Table 1). *System* had the highest number of publications (four), followed by *Journal of Research on technology in Education* (three), *Innovation in Language Learning and Teaching* (three), *TESOL Quarterly* (two), *Australian Journal of Educational Technology* (two), *Education and Information Technologies* (two), and the remaining 15 journals published one article each. Broadly, 87% of the articles were published in the area of Education Educational Research, 38.7% in Linguistics, only one article in Communication and one article in the area of Social Science Other Topics, showing that researchers from all four areas were capturing TEPBLL astounding.

**Table 1.** Journals.

No	Name of SSCI journals	Number of published articles
1	System	4
2	Innovation in Language Learning and Teaching	3
3	Journal of Research on Technology in Education	3
4	Australian Journal of Educational Technology	2
5	Education and Information Technology	2
6	TESOL Quarterly	2
7	Australian Journal of Adult Learning	1
8	Education and Training	1
9	Educational Technology Society	1
10	English for Specific Purposes	1
11	English in Education	1
12	Interactive Learning Environments	1
13	Internet and Higher Education	1
14	RELC Journal	1
15	Research in Science Technological Education	1
16	International Journal of Inclusive Education	1
17	Journal of Computing in Higher Education	1
18	Sage Open	1
19	International Review of Research in Open and Distributed Learning	1
20	Language Learning Technology	1
21	Technical Communication	1
	Total	31

Approximately 61% of the authors were from Asia, followed by the USA, Europe, Australia and Oceania ( Table 1). Among all affiliated regions ( Table 2), the USA and Taiwan invested almost one-fourth of the publications, while China held 16%, demonstrating the significance of these regions’ researchers on the topic. It is important to highlight that this result only reflects the state of empirical research published in SSCI-indexed journals that applied technology-enhanced project-based language learning.

**Table 2.** Authors' affiliations.

Area	Regions	Record count
Asia	Taiwan	8
	China	5
	Japan	2
	South Korea	2
	Thailand	2
	Iran	2
	Malaysia	1
	Oman	1
	Turkey	1
USA	USA	8
Australia	Australia	2
Europe	Spain	3
	Austria	1
	Russia	1
New Zealand	New Zealand	1

### Conceptual frameworks

Further analysis of the results highlighted that the study involved five major categories of theoretical frameworks or concepts, as outlined in Table 3, encompassing social constructivism, sociocultural theory, incidental vocabulary learning, communicative language learning, and self-determination theory. These categories were developed by physically taking on a piece of paper, noting the frameworks or concepts discussed in the papers, and then looking for similar sets. As mentioned earlier, there are two broad classifications of TEPBLL theory: social constructivism and sociocultural theory. According to social constructivism, learners are actively engaged in interactions as developers of knowledge ( Vygotsky, 1980). When social constructivism was proposed, some of the major concepts that fell under this school of thought were virtual reality project-based learning, experiential teaching approaches, digital story-telling projects, collaborative video projects, collaborative digital writing.

**Table 3.** Conceptual framework of research on TEPBLL.

Conceptual frameworks/theories	Research
Social constructivism ○ Virtual reality project-based learning ○ Experiential teaching approach ○ Digital story telling project ○ Collaborative video projects ○ Collaborative digital writing	( Belwal et al., 2021; Chan, 2022; Dooly & Sadler, 2016; Fan, 2024; Garib, 2023; Hsieh et al., 2022; Huang, 2021; Javier Avila-Cabrera & Corral Esteban, 2021; Konieczny & Eckert, 2022; Shi et al., 2024; Tatzl, 2015; Tseng & Yeh, 2019; Wu et al., 2022)
Sociocultural theory ○ Collaborative online learning ○ Collaborative project-based learning ○ Digital video creation ○ Virtual exchange project ○ Scaffolding	( Angeles Mestre-Segarra & Ruiz-Garrido, 2022; Chu et al., 2017; Dugartsyrenova & Sardegna, 2019; Kato et al., 2023; Konieczny & Eckert, 2022; Mohamadi, 2018; Nishioka, 2016; Wu et al., 2022; Yao, 2022; H. C. Yeh & Fu, 2023)
Incidental vocabulary learning	( Avci & Adiguzel, 2017; Tatzl, 2015; H.-C. Yeh et al., 2020)
Communicative language learning	( Avci & Adiguzel, 2017; Dooly & Sadler, 2016; Kato et al., 2023; Owens & Hite, 2022)
Self-determination theory	( Chu et al., 2017; Greenblatt & McDonald, 2022; Tanaka, 2023)

Vygotsky’s social constructivism ( Vygotsky, 1980), which claims that learners participate in knowledge construction through interaction with receiving assistance, was cited most often in the literature. In technology-enhanced project-based language learning, learners used technology — Virtual Reality ( Chan, 2022; Lin & Wang, 2021; Shi et al., 2024; Sun Joo & Lee Jin, 2024), telecollaborative ( Boardman & Hovland, 2022; Fan, 2024; Nami & Asadnia, 2024), digital story telling ( Fan, 2024; Nami & Asadnia, 2024; Nishioka, 2016) — as a tool to interact with the development of the knowledge being pursued as learners engaged each other synchronously and asynchronously. Similarly, when learners were instructed to collaboratively work on a group assignment, they relied reciprocally on each other ( Angeles Mestre-Segarra & Ruiz-Garrido, 2022; Avci & Adiguzel, 2017; Huang, 2021; Owens & Hite, 2022). When two individuals changed their language in search of compliance to cooperate and avoid misunderstanding in case of a breakdown, meaning negotiation occurred when there was repetition of the broken-down meaning, correction, or asking for clarification.

The second most often cited concept was the underlying sociocultural theory, which focused on social and cultural activities and significance of semantic features rather than the formal features of language ( Angeles Mestre-Segarra & Ruiz-Garrido, 2022; Chu et al., 2017; Dugartsyrenova & Sardegna, 2019; Kato et al., 2023; Konieczny & Eckert, 2022; Mohamadi, 2018; Nishioka, 2016; Wu et al., 2022; Yao, 2022; Yeh & Fu, 2023). Some of the constructs under the sociocultural view included supporting technology-enhanced project-based language learning activities, such as collaborative online learning, collaborative project-based learning, digital video creation, virtual exchange project, and scaffolding. In collaborative learning, scaffolding is employed to allow high achievers to guide low achievers in completing complex tasks as a team. This scaffolding occurs in inclusive classrooms: the student with disabilities (SWDs) have the opportunity to feel creative, expand their ideas to global audiences, and increase their literacy skills by collaborating with their peers which is student without disabilities (SWODs) ( Boardman & Hovland, 2022).

The review also showcases three concepts related to language learning: incidental vocabulary learning, communicative language learning, and self-determination theory. The first construct is about incidental vocabulary learning, which implies that learners acquire vocabulary without any prior plan. As Nami & Asadnia (2024) found in her research on digital story telling (DST). She asserts that DST may promote incidental vocabulary acquisition when learners search for or create multimedia resources to illustrate the practical applications of the learned vocabulary or when the word under focus is used in peer-made stories. The second is communicative language learning, which Avci & Adiguzel (2017) involves the concept of authentic material using WhatsApp messenger and has been proven to enhance communicative skills and vocabulary knowledge. The third is self-determination theory, which implies a boost of motivation and self-efficacy from engagement in physical activity, one of which is project-based language learning. Supporting this claim, Chan (2022) proved that the use of virtual reality could positively boost students’ motivation. Overall, most research on TEPBLL has a solid theoretical foundation, and future implications for TEPBLL could combine two or more conceptual frameworks to promote novelty.

### Types of projects and effectiveness of TEPBLL

Theoretically, a strong PBL course is characterized by a cycle of process and product focus, at least partially defined by students, spanning a certain duration that is not limited to a particular class. It also promotes the natural integration of skills: technology and communication skills, are expected to have a dual responsibility of language and content acquisition, ask students to work in groups and also individually, expect students to take some degree of their own learning responsibility through gathering, analyzing, and disseminating of information from target language resources, leading to teachers and students acquiring new roles and responsibilities, delivering a concrete final outcome to a wider populace, and end with the students’ comments on the process and the outcome ( Dressler et al., 2020).

The 31 coded articles had identified seven big umbrellas of project-based language learning type, details exposed in Table 4. Firstly, the total of eight papers applied telecollaborative project. Through this type of project, students could use typical technologies such as *Canvas Website, Wikipedia, e-writingforum, Voxopop, Wimba Voice, Talkshoe, Padlet, Wechat.* All these technologies were employed to support project-based language learning using the Canvas website to trigger communication in STEM ( Owens & Hite, 2022), *Wikipedia* to boost motivation ( Chu et al., 2017) and teching liberal arts ( Konieczny & Eckert, 2022), integration of *Voxopop, Wimba Voice, Talkshoe* ( Dugartsyrenova & Sardegna, 2019) and *WeChat* ( Wu et al., 2022) to enhance intercultural awareness, collaboration between *Facebook, Youtube, Padlet* and *Google classroom* to promote ecological perspective ( Hsieh et al., 2022), *e-writingforum* to support collaborative learning ( Mohamadi, 2018), and *Industrial Tech* to gain engagement during the consultancy project ( Belwal et al., 2021).

**Table 4.** Project type in TEPBLL.

Project type	Name of system	Foci of TEPBLL	Research
Telecollaborative project	Canvas Website Wikipedia e-writingforum Voxopop Wimba Voice Talkshoe Padlet Wechat Industrial tech	Communication in STEM Writing Intercultural Speaking Ecology Engagement	( Belwal et al., 2021; Chu et al., 2017; Dugartsyrenova & Sardegna, 2019; Hsieh et al., 2022; Konieczny & Eckert, 2022; Mohamadi, 2018; Owens & Hite, 2022; Wu et al., 2022)
Digital Story Telling project	SlideShare Inshot iMovie PhotoStory PowerDirector Schoology (LMS)	Vocabulary Content knowledge Reading Writing Speaking Engagement	( Boardman & Hovland, 2022; Fan, 2024; Nami & Asadnia, 2024; Nishioka, 2016)
Virtual reality project	Spherical video-based virtual reality (SVVR) Immersive virtual reality (iVR) Virtual Interpreting Practice (VIP) app Assemblr (AR) Virtual Business Professional (VBP) Google Tour Creator	Creative and innovative thinking Oral English Skill Engagement Motivation Cultural awareness Communication Self-efficacy	( Angeles Mestre-Segarra & Ruiz-Garrido, 2022; Chan, 2022; Lin & Wang, 2023; Owens & Hite, 2022; Shi et al., 2024; Sun Joo & Lee Jin, 2024)
Video making Project	Google Docs EverCam PowerDirector Movie Maker YouTube Web 2.0	Writing Grammar Vocabulary Speaking Writing (translation)	( Garib, 2023; Greenblatt & McDonald, 2022; Nishioka, 2016; H. C. Yeh, 2018; H. C. Yeh & Fu, 2023)
Mobile instant messaging-based Project	Online discussion forum WeChat Whatsapp	Climate education Intercultural Communication Vocabulary	( Avci & Adiguzel, 2017; Nami & Asadnia, 2024; Yao, 2022)
Video Conferencing Project	Machinima Skype	Instil Habitual Communication Intercultural	( Dooly & Sadler, 2016; Kato et al., 2023)
Audio-visual Translation project	SubESPSKills Moodle	Listening Writing Dubbing	( Javier Avila-Cabrera & Corral Esteban, 2021)

Second, three studies applied the digital story telling project (DST). Boardman & Hovland (2022) could engage student with disabilities through DST using the notion of scaffold learning with their SWOD peers. He consented to the literacy skills and found that SWD could expand their ideas and express their creativity through collaboration with their peers. Further, Nami & Asadnia (2024) adopting the multimodalities to create DST, she combines animated DS, Game-based DS, Social media DS, Synchronous collaborative DS and Micro DS to enhance incidental and intentional vocabulary learning. Fan (2024) asserted that DST can facilitate content knowledge, language proficiency and academic competence. In the context of medical-related majors, Fan explored DST to encourage students to develop content related to their context, composing a written text, making an oral script, and doing a voice over to their video presentation. The final result showed that students were more aware of rhetorical, linguistic and inter-semiotic choices.

Third, six studies focused on a virtual reality project employing *Spherical video-based virtual reality (SVVR), Immersive virtual reality (iVR), Virtual Interpreting Practice (VIP) app, Assemblr (AR), Virtual Business Professional (VBP), Google Tour Creator.* *Spherical video-based virtual reality* (SVVR) allows students to co-create content for travel books. Students possibly choose content and create a layout for their written work and then uploaded to YouTube, this kind of activity is empirically foster creativity and curiosity ( Sun Joo & Lee Jin, 2024). Similarly, *Immersive virtual reality* (iVR) was used to enhance students’ oral English skills and engagement. Shi et al. (2024) employed iVR to create VR videos by inputting the dialogue script, building the dialogue setting, sharing it through the VR platform, practicing and recording the dialogue into the VR system. Another study on VR involved *Virtual Interpreting Practice* (VIP) application to foster interpreting skills. Another system called *Assemblr* (AR) to design a three-fold pamphlet QR code contains 3D interactive content of university tours for freshmen. Lin & Wang (2023) indicated the effect of using AR was on learner’s perception of creativity and boosted their motivation towards learn with technology. Thereupon, *Virtual Business Professional* (VBP) was employed in the study of Angeles Mestre-Segarra & Ruiz-Garrido (2022) and empirically promoted language-content communication and fostered intercultural awareness through collaborative online international learning. The last system under review is *Google Tour Creator.* Lin & Wang (2021) assigned participants to create virtual tours platform to introduce their hometown to an international student. This study confirmed that the VR project could facilitate students’ efficacy in creative thinking, and that technical skills also benefit their English communication skills.

Fourth, the gaining attention in TEPBLL area is conducting video-making project. Five studies employed TEPBLL with the aid of *Google Docs, EverCam, PowerDirector, Movie Maker, YouTube,* and *Web 2.0* platform to their project. The collaboration between *Movie Maker* and *Google Docs* empirically enhanced students’ communicative performance by creating public service announcement (PSA) videos ( Greenblatt & McDonald, 2022). Nishioka (2016) conducted research on collaborative DST using a Web 2.0 based application. The results showed that learners strategically used their first language, grammatical terminology, and private speech in the collaborative knowledge construction process during the project. To promote intra-cultural understanding, Yeh & Fu (2023) research has been conducted on video creation using *Power Director* which implies the improvement of English communication and deepens cultural understanding.

Fifth, three studies on mobile instant messaging-based projects employed *online discussion forum*, *WeChat*, and *Whatsapp* platforms. The study of Nami & Asadnia (2024) used a multimodal platform of *Whatsapp, slideshare, blogpage, inshot* and *Instagram* to implement word contextualization, and story presentation through a learner-generated digital story telling (DST) project. The results indicated that DST design, development, presentation, and evaluation positively enhanced learners’ perceptions towards DST-based vocabulary learning. Avci & Adiguzel (2017) further researched the use of *Whatsapp* to foster communication skills and found that mobile instant messaging had a positive effect on student performance. Yao (2022) conducted research on climate education using online discussion forums, which increased learners’ willingness to combat climate change. Sixth, the studies focus on the Video Conferencing project with the aid of *Machinima* ( Dooly & Sadler, 2016) and *Skype* ( Kato et al., 2023) platform, focusing on speaking skills. The last is an Audio-visual Translation project employs *SubESPSKills* and *Moodle.* Javier Avila-Cabrera & Corral Esteban (2021) proposed *SubESPSKills* innovative project on subtitling tasks in an English for Specific Purposes class to improve written production skills. The results indicated improvements in writing production skills and some language knowledge (e.g., words, structure, etc.). Furthermore, the language skill and knowledge along with student and teacher activities of TEPBLL also portrayed in Table 5.

**Table 5.** Language skills and technologies in TEPBLL.

Language skills and knowledge	Technologies	Student activities	Teacher activities
Speaking	Audio Visual Translation tool (AVT)	Listening task, writing essays, reading exercises and creative dubbing assignment.	Supervising and checking the project process
	iMovie	Students take part in 3-minute group video assignment that were associated to the learning context of their classroom textbook.	Offering assistance when necessary
	Immersive Virtual Reality (IVR)	Create VR video using presentation, practice, and production procedure.	Supervise and check the project process
	Virtual Interpreting Practice (VIP)	Students is given an interpreting task. Each task (source text/audio/video) is 2 until 8 minutes long or about 250–350 words/400–700 characters.	Teacher played the audio files of pre-recorded speeches for in-class practice and monitoring student activity
	Google Classroom	Content development, written text, multimodal elements, oral script and voice over narration, tech training, presentation.	Lead group discussion, guide students
	Skype	Dual nations students made pairs and communicate.	Monitoring
Listening	Audio Visual Translation tool (AVT)	Listening comprehension exercises, composing reverse subtitle for assigned video.	Not stated
Reading	Moodle	Reading exercise for reverse subtitling.	Explaining the use of Moodle
Writing	Wikipedia	Enhance an already-existing Wikipedia page or write a new one about course-related subject.	Assess Wikipedia articles published by students
	Audio Visual Translation tool (AVT)	Listening comprehension exercises, composing reverse subtitle for assigned video.	Organizing stages for project activities
	Google Tour Creator	Student created virtual tours using Google Tour Creator to showcase their hometown to a global audience.	Guide student to do the project
Vocabulary	WhatsApp/Telegram	Discussing through chat on designing multi-modal digital story, development, and presentation.	Reducing group conflict
Creative and Critical Thinking	Spherical video-based virtual reality (SVVR)	While using SVVR technology to produce online multimedia to complement their written work – which would then be posted on YouTube – groups were required to select content and design layout for their chapter first.	Make notes, recorded writing progress each group, provide feedback
	Google tour creator (VR)	Create multiple VR scenes and create a tour on the desktop with Google’s street-view technology.	Guide student to do the project
Intercultural Awareness	WeChat	Students were encouraged to use English to communicate their ideas about any topics based on their interest.	Make a group on WeChat and give comment
	PowerDirector	Create video introducing their local cultures.	Correcting student’s error
	Padlet and Facebook	Student-produced presentation slides and videos doing joint project related to SDGs.	Supervising the collaboration process
Content Knowledge	Canvas	Students collaborated to build a model of the water cycle and its potential relationship to air pollution. Second, students composed/write a response to the problem.	Established and presented the project and driving question were established

Overall, from the above results, it can be seen that the learners had an overall positive attitude towards TEPBLL and their learning outcomes were also satisfactory. The review results in the same nuance support Dressler et al. (2020) and Casal & Bikowski (2020) findings about the benefit of project-based language learning using technology that improves language skills (speaking, listening, writing, reading, vocabulary acquisition), increase intercultural awareness, and triggers creative and critical thinking.

### Implications of TEPBLL

Based on the analysis the 31 articles, we classified and briefly summarized the articles from three dimensions: language acquisition, teacher’s roles, and potential research on TEPBLL. In the language acquisition dimension, the previously mentioned literature showed that task design and students’ technological pedagogical content knowledge had great impacts on TEPBLL. Teachers and coordinators in the language program perceived that the content being taught has implications for student motivation in learning language ( Nanni, 2021), higher levels of motivation and acquisition of relevant skills ( Konieczny & Eckert, 2022), and expanding learning experiences and effectiveness, making learning a joyful and pervasive process, improving learners’ autonomy, and critical thinking, and increasing their motivation and self-confidence ( Chan, 2022).

In the teacher roles dimension, the literature indicates that teachers are urged to inaugurate TEPBLL activities by describing technological knowledge and learning objectives to students. As for teacher facilitation, it is also recommended that the teacher should fully understand their students’ perception of language proficiency to set up content and language-related objectives ( Sun Joo & Lee Jin, 2024) the same nuance asserted by Jao et al. (2023) that the digital story-telling has its level, so that the teacher should assign the project by their level of language proficiency. Another recommendation is that teachers present practical tutorials and provide fruitful tips for the project. Moreover, teachers should assign specific roles and responsibilities to each group member to ensure everyone participates ( Huang, 2021), the teacher sets up staged project tasks and provides progressive feedback, since real-time corrective feedback sustains learner motivation.

Additionally, this review asserts five potential topics for further exploration of future research: (1) the exploration of potential technology used for TEPBLL that suites inclusive classrooms ( Boardman & Hovland, 2022); (2) possible influences of students’ emotional states on their project performance ( Shi et al., 2024); (3) facilitating content knowledge and language proficiency using robots ( Fan, 2024); (4) explore the challenges and limitations faced by students in the process of collaborative video-making to refine the process ( Yeh et al., 2020); and (5) a higher number of students may help to generalize the results ( Mohamadi, 2018).

## Discussion

The review results indicated that certain studies have integrated technology tools into PBLL and confirmed that creative student output is unattainable through conventional courses. The incorporation of technology into project-based language learning was the principal factor contributing to the efficacy of TEPBLL in enhancing language knowledge, as it heightened students’ engagement and intrinsic motivation, stimulated creative and critical thinking, fostered collaboration, refined essential communication skills, and exposed intercultural understanding, ultimately leading to successful learning outcomes ( Avci & Adiguzel, 2017; Garib, 2023; Greenblatt & McDonald, 2022; Hsieh et al., 2022; Nami & Asadnia, 2024). The results of our review indicated that the majority of learners expressed positive attitudes towards TEPBLL ( H.-W. Huang, 2021; Javier Avila-Cabrera & Corral Esteban, 2021; Lin & Wang, 2021).

Furthermore, technology (i.e., Virtual Reality) also regarded as a rejuvenating and relaxing learning experience that benefited students’ English skills, as the project was engaging and enabled autonomy. Although it is noteworthy that students with high creative self-efficacy tend to feel less pressure than those with low creative self-efficacy, the result of their EMI aspect (i.e. enjoyment, perceive competence, perceived choice, usefulness), yet the post-test score showed no difference ( Lin & Wang, 2021). In this way, technology-enhanced PBLL (i.e. immersive VR) can promote social engagement by participating and interacting in the VR environment with their peers ( Shi et al., 2024).

It was also found that TEPBLL could enrich subject-specific vocabulary ( Dugartsyrenova & Sardegna, 2019; Javier Avila-Cabrera & Corral Esteban, 2021; Tatzl, 2015), resulting in active engagement in communicative activities among students with high degree of autonomy and motivation ( Greenblatt & McDonald, 2022). Since PBLL offers a hands-on approach to a real work-life situations, virtual interpreting practice (VIP) allows students to experience and practice their interpreting competency in authentic and immersive interpreting practice. Moreover, students generally had a positive attitude towards VIP for interpreting learning ( Chan, 2022).

As new and more inventive educational technologies are constantly being developed and replacing those that are now in use, our goal is not to propose a list of technologies for future use. Our primary goal is to provide a general overview of the types of technology that can be used in cooperative language learning activities to promote different language knowledge and skills. The review findings demonstrated the beneficial effects of AVT, iMovie, Immersive VR, Virtual Interpreting Practice (VIP) tools, and Skype in enhancing students’ speaking skills through TEPBLL. As defined in the literature, these technologies enable individuals to improve their speaking skills by fostering interactions, and offering socialized interactive environments as presented in Table 5.

Despite these positive results reflecting the impact of TEPBLL on language acquisition, at least two studies indicated subpar performance of participants in enhancing technology in PBLL. Mohamadi (2018) conducted a comparative study of PBL and e-PBL of English idiom knowledge and found that students faced a challenge in using technology because of the quality of the Internet, which caused them to not enjoy the same benefits as the non-tech group. Nishioka (2016) investigated a Web 2.0-based collaborative digital story-telling project but found that most proficient learners showed dissatisfaction in collaborating with less proficient learners, as it took a longer time to complete the task. However, Nishioka also suggested that language educators could boost the motivation and engagement of highly proficient learners by highlighting how collaborative learning with peers facilitates the acquisition of the target language within the project. Future research should further investigate these issues and identify the most suitable approach to assist.

## Conclusion

Overall, we examined 31 SSCI articles and found five conceptual frameworks in TEPBLL area that covered social constructivism theory, sociocultural theory, incidental vocabulary learning, communicative language learning and self-determination theory. Seven project types involving telecollaborative project, digital story-telling, virtual reality, video making, mobile instant messaging-based, video conferencing and audio-visual translation projects were promoted after the enhancement of TEPBLL in the classroom. Thus, eight language skills and knowledge would be improved after the application of TEPBLL: speaking skills, listening skills, reading skills, writing skills, vocabulary knowledge, creative and critical thinking skills, intercultural awareness, and content knowledge. We also discussed the recommendations of previous studies in detail and considered possible research topics for future research.

Thus, it is crucial to note that this review focused on identifying a limited number of empirical works that employed technology to support the process of language learning and that were published in SSCI; nevertheless, several significant publications on TEPBLL were not covered in this review. For example, Dressler et al. (2020) integrated content and language learning in project-based learning implies the concept of CLIL-infused TEPBLL, which supports vocational or content-related majors conducting PBLL. However, the review foci are the theories that were used in the empirical studies on TEPBLL or the technologies aimed at language enhancement, and the implications for future research. However, there are certain limitations of this review that might be considered in the following ways in future research: (1) The current study used a limited number of articles from a single resource, that is Web of Science, to maintain the quality of the review. Therefore, it is recommended that future studies cover multiple resources such as Google Scholar, IEEE Exploration and other electronic archives, including conference papers, book chapters, and publications in non-SSCI journals; (2) Future studies may consider analyzing the studies from varied pedagogical perspectives.

## Ethics and consent

No ethics and consent were required.

## Data Availability

Dataset of Systematic Literature Review of Technology Enhanced Project-based Language Learning at https://doi.org/10.5281/zenodo.15792672 ( Fikria et al., 2025a) PRISMA Checklist for ‘A Systematic Review of Technology-Enhanced Project-Based Language Learning: Theoretical Frameworks, Project Types, and Implications for Future Research and Practice’. https://doi.org/10.5281/zenodo.15792956 ( Fikria et al., 2025b) Data associated with this article is provided in online repository under the terms of Creative Common Attribution 4.0 International (CC BY 4.0).
