# A Systematic Review of Technology-Enhanced Project-Based Language Learning: Theoretical Frameworks, Project Types, and Implications for Future Research and Practice

**DOI:** 10.12688/f1000research.165491.2

**Published:** 2025-12-27

**Authors:** Ainun Fikria, Slamet Setiawan, Ahmad Munir

**Affiliations:** 1 Language and Literature Education, Universitas Negeri Surabaya, Surabaya, East Java, Indonesia

**Keywords:** Project-based language learning, educational technology, computer assisted language learning, technology enhanced language learning, systematic literature review.

## Abstract

**Background:**

The integration of digital technologies into project-based language learning has attracted growing scholarly attention; however, research in this area remains conceptually fragmented. A systematic synthesis of theoretical foundations, projects typologies, language skills and knowledge addressed, and pedagogical implications of technology-enhanced project-based language learning (TEPBLL) is still limited.

**Methods:**

Restricted to SSCI-indexed publications and guided by PRISMA 2020 protocols, this systematic review examined 31 empirical studies published between 2015 and 2024. The analysis focused on the nature of publications, underlying theoretical frameworks, types of technology-supported projects, reported language-learning outcomes, and implications for research and instructional practice.

**Results:**

The reviewed studies indicate that TEPBLL research is predominantly grounded in five theoretical frameworks and encompasses seven diverse technology-supported projects types. Across studies, TEPBLL is frequently reported improvements in eight major skills and knowledge areas. The review also identifies recurring three dimensions of implications for instructional practice and future research.

**Conclusions:**

This review systematically synthesizes prevailing patterns in the TEPBLL literature. The study offers a coherent and comprehensive overview that can guide future research design and support more theory-informed, methodologically rigorous, and context-sensitive application of TEPBLL.

## Introduction

Technology-enhanced learning has been a persistent topic in education as it engages students ( Munaro et al., 2021) and encourages teachers in designing innovative learning environments ( Garib, 2023). However, while technology is widely adopted in educational contexts, its application does not always lead to meaningful learning, particularly in language education ( Lee, 2022). In response to this issue, Project-based language learning (PBLL) — a constructivist, student-centered approach — has emerged as a promising framework that emphasizes real-world, authentic projects to foster deeper learning.

Within PBLL, the integration of technology has the potential to enhance engagement, foster collaboration, and promote the practical use of language in authentic contexts. Prior research has explored various aspects of technology-enhanced language learning (TELL) ( Ahmadi, 2018; Golonka et al., 2012), and studies have examined how tools like virtual reality, chatbots, and adaptive learning systems can support language acquisition ( Bahari, 2024; Palanisamy, 2024). Yet, these studies are often fragmented, focusing on individual tools or isolated pedagogical outcomes without offering a consolidated understanding of how technology and PBLL intersect.

Although individual technologies and language teaching approaches have been extensively studies, there is a lack of comprehensive analysis that systematically investigates how technology-enhanced project-based language learning (TEPBLL) is being implemented, what technologies are being used, what skills are being developed, and which theoretical frameworks underpin these practices. This absence of synthesized overview limits educators and researchers the ability to make informed decisions about the effective integration of technology into PBLL contexts.

Despite the rapid increase of TELL and PBLL studies, the intersection of the two domains remains underexplores. Existing research tends to examine individual technologies or isolated pedagogical outcomes rather than providing an integrated understanding of how technology is operationalized within PBLL context. Despite the rapid growth of technology-enhanced language learning and project-based pedagogy, no prior systematic review to date has comprehensively mapped the theoretical foundations, project typologies, technological tools, language-learning outcomes, and pedagogical implications of TEPBLL over the past decade. To address this gap, this review is guided by the following research questions:

(1) What is the nature of published research on TEPBLL?
(2) What theoretical frameworks underpin TEPBLL research?
(3) What types of projects and technologies are used in TEPBLL practices?
(4) What language skills or knowledge areas are reported to be enhanced through TEPBLL?
(5) What implications for research and practice emerge from existing studies?

### The concept of technology-enhanced project based language learning

Technology-enhanced project-based language learning (TEPBLL) is an innovative approach to education that incorporates the use of projects in learning a second language and technology in the authentic use of the second and/or foreign language. This approach involves the use of modern technology tools and platforms to complete project-based assignments that involve the use of oral and written language, innovatively created to enable technical, collaborative, and real-life assignments ( Dooly et al., 2021; Syzenko & Diachkova, 2020). TEPBLL consequently delineates the possibilities for student engagement in 21st-century knowledge, skills, and competencies in project-based language learning through technology-enhanced tasks and activities that enhance learning experiences ( Dooly et al., 2021). In PBLL, the project’s objective is authentic and the target audience for its result is real because it is relevant to circumstances outside of the classroom.

This enables educators to develop engaging learning environments that enhance students’ creative thinking, innovation, problem-solving abilities, and communication skills ( Sun & Yang, 2013; Syzenko & Diachkova, 2020). As a theory, TEPBLL holds that learners are more likely to acquire a second language when such learning is a by-product of the need to use a target language for meaningful tasks and purposes. Project-based language learning via technology, namely TEPBLL, allows for the effective use of technology in facilitating language acquisition which can enable students to achieve efficient verbal communication in real-life situations, as supported by Nur et al. (2022) and Syzenko & Diachkova (2020). Overall, TEPBLL is a teaching strategy that integrates technology into project-based learning environments, aimed at effectively and meaningfully improving students’ language abilities, critical thinking skills and digital literacy.

## Methods

This study adhered to a three-stage procedure consisting of literature research, article screening, and data analysis, in accordance with PRISMA 2020 checklist guidelines ( Fikria et al., 2025c).

### Search strategy

The Web of Science (WoS) database was selected because of its rigorous indexing standards and wide coverage of SSCI journals, following prior systematic reviews (e.g. Su & Zou, 2020; Zou et al., 2019), the search was restricted to SSCI-indexed empirical research to ensure quality and methodological transparency.

The exact search string used in Web of Science with Boolean search syntax, was TS = ((“project-based language learning” AND technology) AND (“English language learning” OR EFL)). Filters were applied to include SSCI journal articles published between 2015 and 2024. The search was conducted in November 2024, yielding 319 records.

### Inclusion and exclusion criteria

**Figure 1.**
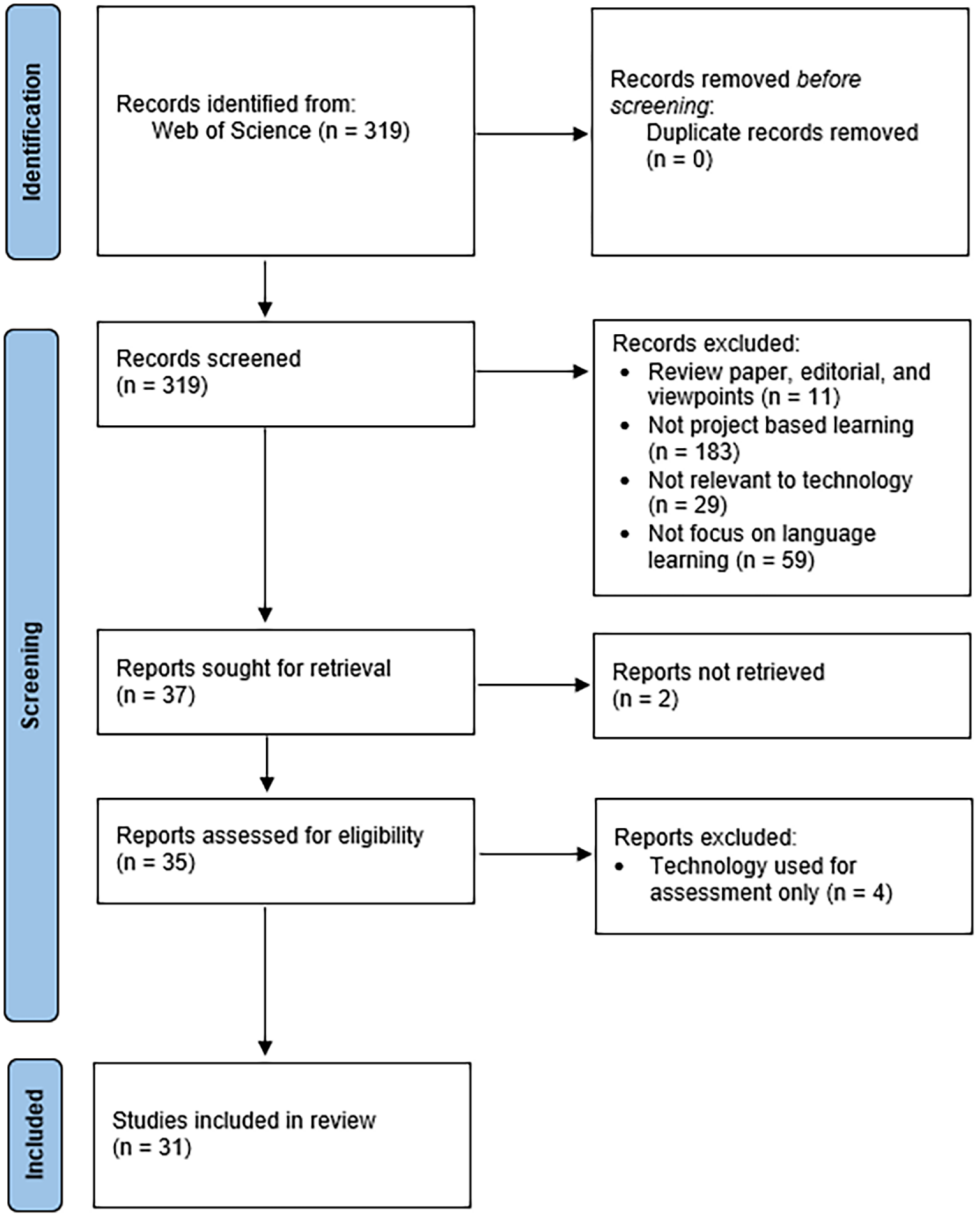
PRISMA 2020 flow diagram.

Four gates of exclusion criteria were applied to ensure relevance to TEPBLL. Articles were excluded if they were: a review articles, editorials, and viewpoints; unrelated to project-based language learning; not employing any technology within the PBLL framework; using technology solely for automated scoring or assessment. We included articles that: were empirical studies, integrated technology into project-based language learning, and provided clear description of the technology and learning activity used. After removing ineligible items across all screening stages, 31 studies met all criteria and were included in the final analysis. The PRISMA flow diagram ( Figure 1) summarizes the identification, screening, and inclusion process.

### Coding process and data synthesis

A bottom-up approach was adopted to ensure an inductive and data-driven analysis. Two researchers independently reviewed the 31 articles and coded them using five guiding questions developed for this review. The coding process involved three process: initial extraction, grouping by similarity, iterative refinement. Initial extraction involved identifying and summarizing relevant information from each study, including theoretical frameworks, project types, technologies used, targeted language skills, and reported implications. Grouping by similarity was then carried out by categorizing the extracted data according to conceptual resemblance, supported by the creation of dataset tables for systematic comparison ( Fikria et al., 2025a). Finally, iterative refinement was conducted through repeated discussions among the researchers to review, adjust, validate the coding categories until consensus was achieved regarding category definitions and interpretations.

To ensure reliability, two researchers mutually coded three articles selected at random and then discussed and compared codes and came down to fine-tune the articles until both readers had a similar understanding of the selected articles as well as the coding frame work before they were allowed to self-code the rest of the selected articles. In this process, both the researchers always remembered that which we are going to code should be the actual meanings of the articles and then they read the articles again where they simply summarized what those articles described in terms of the theoretical frameworks, technologies, research findings and implications and then only coded them. Subsequently, the labels were grouped and categorized into groups of similarity and subdivided as necessary with extra caution. For example, create VR video using presentation, practice, and production procedure ( Shi et al., 2024), students determine their own project, collected data, and discussed the data ( Hsieh et al., 2022), students also synthesized data and conclusions ( Tanaka, 2023) were coded as a ‘student activity’. If there was any disagreement regarding some aspect of the data, an analysis of the results was conducted by discussion with the third researcher. Interrater reliability was calculated using Cohen’s kappa (κ), yielding κ = 0.808 ( Fikria et al., 2025b), 95% CI = 0.70–0.93, indicating substantial to almost perfect agreement ( Landis & Koch, 1977).

Due to heterogeneity across the studies—particularly in research designs, outcome measures, assessment tools, and reporting standards—a meta-analysis was not feasible. Only a small number of studies (n = 6) reported statistical information that could be converted into standardized effect sizes, and reporting was inconsistent. Therefore, a descriptive and narrative synthesis approach was adopted to summarize: the nature of TEPBLL publications; the theoretical frameworks applied; the project types and technologies utilized; the language skills and knowledge addressed, and the pedagogical implications reported.

## Results

The findings section consists of the nature of published research on TEPBLL, exploring the framework theories covering TEPBLL, the type of projects used in TEPBLL, type of language knowledge and skills that could be promoted by TEPBLL, implications derived upon the application of TEPBLL, and discussion on the steps involved in conducting TEPBLL in all contexts. The flow chart in Figure 1 portrays the research and selection of eligible studies. The initial research generated total of 319 articles. After checking duplication, it remains 319 articles to be identified for title and abstract screening. 37 full-text were included for further analysis. 31 of them met the eligibility criteria that were included in the study. Due to high heterogeneity and incomplete reporting of quantitative metrics, a meta-analysis was not attempted. Instead, descriptive and narrative synthesis approach was used to summarize research trends.

### The nature of published research on TEPBLL

**Figure 2.**
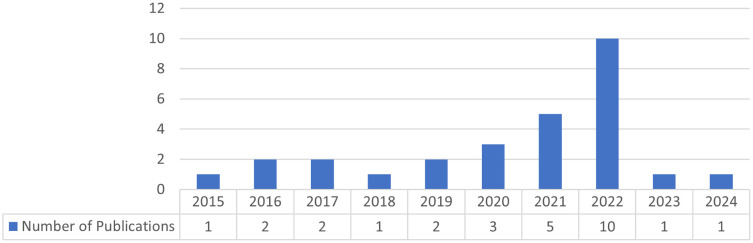
Publication date.

Research in technology enhanced project-based language learning has been gaining a stable trend in past decades, with a notable increase of publication in 2022 ( Figure 2).

The 31 studies was published across 21 SSCI-indexed journals, with System publishing the highest number (four articles), followed by *Journal of Research on technology in Education* and *Innovation in Language Learning and Teaching* (three each). Most studies (87%) were categorized under Education Educational research, followed by Linguistics (38.7%), with a few scattered across Communication and other Social Science domains. This trends showed that researchers from all areas were capturing TEPBLL astounding ( Table 1).

**Table 1.** Journals.

No	Name of SSCI journals	Number of published articles
1	System	4
2	Innovation in Language Learning and Teaching	3
3	Journal of Research on Technology in Education	3
4	Australian Journal of Educational Technology	2
5	Education and Information Technology	2
6	TESOL Quarterly	2
7	Australian Journal of Adult Learning	1
8	Education and Training	1
9	Educational Technology Society	1
10	English for Specific Purposes	1
11	English in Education	1
12	Interactive Learning Environments	1
13	Internet and Higher Education	1
14	RELC Journal	1
15	Research in Science Technological Education	1
16	International Journal of Inclusive Education	1
17	Journal of Computing in Higher Education	1
18	Sage Open	1
19	International Review of Research in Open and Distributed Learning	1
20	Language Learning Technology	1
21	Technical Communication	1
	Total	31

Approximately 61% of the authors were from Asia, followed by the USA, Europe, Australia and Oceania ( Table 1). Among all affiliated regions ( Table 2), the USA and Taiwan invested almost one-fourth of the publications, while China held 16%, demonstrating the significance of these regions’ researchers on the topic. It is important to highlight that this result only reflects the state of empirical research published in SSCI-indexed journals that applied technology-enhanced project-based language learning.

**Table 2.** Authors' affiliations.

Area	Regions	Record count
Asia	Taiwan	8
	China	5
	Japan	2
	South Korea	2
	Thailand	2
	Iran	2
	Malaysia	1
	Oman	1
	Turkey	1
USA	USA	8
Australia	Australia	2
Europe	Spain	3
	Austria	1
	Russia	1
New Zealand	New Zealand	1

### Conceptual frameworks

Further analysis of the results highlighted that the study involved five major categories of theoretical frameworks or concepts, as outlined in Table 3, encompassing social constructivism, sociocultural theory, incidental vocabulary learning, communicative language learning, and self-determination theory. These categories were developed by physically taking on a piece of paper, noting the frameworks or concepts discussed in the papers, and then looking for similar sets. As mentioned earlier, there are two broad classifications of TEPBLL theory: social constructivism and sociocultural theory. According to social constructivism, learners are actively engaged in interactions as developers of knowledge ( Vygotsky, 1980). When social constructivism was proposed, some of the major concepts that fell under this school of thought were virtual reality project-based learning, experiential teaching approaches, digital story-telling projects, collaborative video projects, collaborative digital writing.

**Table 3.** Conceptual framework of research on TEPBLL.

Conceptual frameworks/theories	Research
Social constructivism ○ Virtual reality project-based learning ○ Experiential teaching approach ○ Digital story telling project ○ Collaborative video projects ○ Collaborative digital writing	( Belwal et al., 2021; Chan, 2022; Dooly & Sadler, 2016; Fan, 2024; Garib, 2023; Hsieh et al., 2022; Huang, 2021; Javier Avila-Cabrera & Corral Esteban, 2021; Konieczny & Eckert, 2022; Shi et al., 2024; Tatzl, 2015; Tseng & Yeh, 2019; Wu et al., 2022)
Sociocultural theory ○ Collaborative online learning ○ Collaborative project-based learning ○ Digital video creation ○ Virtual exchange project ○ Scaffolding	( Angeles Mestre-Segarra & Ruiz-Garrido, 2022; Chu et al., 2017; Dugartsyrenova & Sardegna, 2019; Kato et al., 2023; Konieczny & Eckert, 2022; Mohamadi, 2018; Nishioka, 2016; Wu et al., 2022; Yao, 2022; H. C. Yeh & Fu, 2023)
Incidental vocabulary learning	( Avci & Adiguzel, 2017; Tatzl, 2015; H.-C. Yeh et al., 2020)
Communicative language learning	( Avci & Adiguzel, 2017; Dooly & Sadler, 2016; Kato et al., 2023; Owens & Hite, 2022)
Self-determination theory	( Chu et al., 2017; Greenblatt & McDonald, 2022; Tanaka, 2023)

Vygotsky’s social constructivism ( Vygotsky, 1980), which claims that learners participate in knowledge construction through interaction with receiving assistance, was cited most often in the literature. In technology-enhanced project-based language learning, learners used technology — Virtual Reality ( Chan, 2022; Lin & Wang, 2021; Shi et al., 2024; Sun Joo & Lee Jin, 2024), telecollaborative ( Boardman & Hovland, 2022; Fan, 2024; Nami & Asadnia, 2024), digital story telling ( Fan, 2024; Nami & Asadnia, 2024; Nishioka, 2016) — as a tool to interact with the development of the knowledge being pursued as learners engaged each other synchronously and asynchronously. Similarly, when learners were instructed to collaboratively work on a group assignment, they relied reciprocally on each other ( Angeles Mestre-Segarra & Ruiz-Garrido, 2022; Avci & Adiguzel, 2017; Huang, 2021; Owens & Hite, 2022). When two individuals changed their language in search of compliance to cooperate and avoid misunderstanding in case of a breakdown, meaning negotiation occurred when there was repetition of the broken-down meaning, correction, or asking for clarification.

The second most often cited concept was the underlying sociocultural theory, which focused on social and cultural activities and significance of semantic features rather than the formal features of language ( Angeles Mestre-Segarra & Ruiz-Garrido, 2022; Chu et al., 2017; Dugartsyrenova & Sardegna, 2019; Kato et al., 2023; Konieczny & Eckert, 2022; Mohamadi, 2018; Nishioka, 2016; Wu et al., 2022; Yao, 2022; Yeh & Fu, 2023). Some of the constructs under the sociocultural view included supporting technology-enhanced project-based language learning activities, such as collaborative online learning, collaborative project-based learning, digital video creation, virtual exchange project, and scaffolding. In collaborative learning, scaffolding is employed to allow high achievers to guide low achievers in completing complex tasks as a team. This scaffolding occurs in inclusive classrooms: the student with disabilities (SWDs) have the opportunity to feel creative, expand their ideas to global audiences, and increase their literacy skills by collaborating with their peers which is student without disabilities (SWODs) ( Boardman & Hovland, 2022).

The review also showcases three concepts related to language learning: incidental vocabulary learning, communicative language learning, and self-determination theory. The first construct is about incidental vocabulary learning, which implies that learners acquire vocabulary without any prior plan. As Nami & Asadnia (2024) found in her research on digital story telling (DST). She asserts that DST may promote incidental vocabulary acquisition when learners search for or create multimedia resources to illustrate the practical applications of the learned vocabulary or when the word under focus is used in peer-made stories. The second is communicative language learning, which Avci & Adiguzel (2017) involves the concept of authentic material using WhatsApp messenger and has been proven to enhance communicative skills and vocabulary knowledge. The third is self-determination theory, which implies a boost of motivation and self-efficacy from engagement in physical activity, one of which is project-based language learning. Supporting this claim, Chan (2022) proved that the use of virtual reality could positively boost students’ motivation. Overall, most research on TEPBLL has a solid theoretical foundation, and future implications for TEPBLL could combine two or more conceptual frameworks to promote novelty.

### Types of projects and effectiveness of TEPBLL

Theoretically, a strong PBL course is characterized by a cycle of process and product focus, at least partially defined by students, spanning a certain duration that is not limited to a particular class. It also promotes the natural integration of skills: technology and communication skills, are expected to have a dual responsibility of language and content acquisition, ask students to work in groups and also individually, expect students to take some degree of their own learning responsibility through gathering, analyzing, and disseminating of information from target language resources, leading to teachers and students acquiring new roles and responsibilities, delivering a concrete final outcome to a wider populace, and end with the students’ comments on the process and the outcome ( Dressler et al., 2020).

The 31 coded articles had identified seven big umbrellas of project-based language learning type, details exposed in Table 4. Firstly, the total of eight papers applied telecollaborative project. Through this type of project, students could use typical technologies such as *Canvas Website, Wikipedia, e-writingforum, Voxopop, Wimba Voice, Talkshoe, Padlet, Wechat.* All these technologies were employed to support project-based language learning using the Canvas website to trigger communication in STEM ( Owens & Hite, 2022), *Wikipedia* to boost motivation ( Chu et al., 2017) and teching liberal arts ( Konieczny & Eckert, 2022), integration of *Voxopop, Wimba Voice, Talkshoe* ( Dugartsyrenova & Sardegna, 2019) and *WeChat* ( Wu et al., 2022) to enhance intercultural awareness, collaboration between *Facebook, Youtube, Padlet* and *Google classroom* to promote ecological perspective ( Hsieh et al., 2022), *e-writingforum* to support collaborative learning ( Mohamadi, 2018), and *Industrial Tech* to gain engagement during the consultancy project ( Belwal et al., 2021).

**Table 4.** Project type in TEPBLL.

Project type	Name of system	Foci of TEPBLL	Research
Telecollaborative project	Canvas Website Wikipedia e-writingforum Voxopop Wimba Voice Talkshoe Padlet Wechat Industrial tech	Communication in STEM Writing Intercultural Speaking Ecology Engagement	( Belwal et al., 2021; Chu et al., 2017; Dugartsyrenova & Sardegna, 2019; Hsieh et al., 2022; Konieczny & Eckert, 2022; Mohamadi, 2018; Owens & Hite, 2022; Wu et al., 2022)
Digital Story Telling project	SlideShare Inshot iMovie PhotoStory PowerDirector Schoology (LMS)	Vocabulary Content knowledge Reading Writing Speaking Engagement	( Boardman & Hovland, 2022; Fan, 2024; Nami & Asadnia, 2024; Nishioka, 2016)
Virtual reality project	Spherical video-based virtual reality (SVVR) Immersive virtual reality (iVR) Virtual Interpreting Practice (VIP) app Assemblr (AR) Virtual Business Professional (VBP) Google Tour Creator	Creative and innovative thinking Oral English Skill Engagement Motivation Cultural awareness Communication Self-efficacy	( Angeles Mestre-Segarra & Ruiz-Garrido, 2022; Chan, 2022; Lin & Wang, 2023; Owens & Hite, 2022; Shi et al., 2024; Sun Joo & Lee Jin, 2024)
Video making Project	Google Docs EverCam PowerDirector Movie Maker YouTube Web 2.0	Writing Grammar Vocabulary Speaking Writing (translation)	( Garib, 2023; Greenblatt & McDonald, 2022; Nishioka, 2016; H. C. Yeh, 2018; H. C. Yeh & Fu, 2023)
Mobile instant messaging-based Project	Online discussion forum WeChat Whatsapp	Climate education Intercultural Communication Vocabulary	( Avci & Adiguzel, 2017; Nami & Asadnia, 2024; Yao, 2022)
Video Conferencing Project	Machinima Skype	Instil Habitual Communication Intercultural	( Dooly & Sadler, 2016; Kato et al., 2023)
Audio-visual Translation project	SubESPSKills Moodle	Listening Writing Dubbing	( Javier Avila-Cabrera & Corral Esteban, 2021)

Second, three studies applied the digital story telling project (DST). Boardman & Hovland (2022) could engage student with disabilities through DST using the notion of scaffold learning with their SWOD peers. He consented to the literacy skills and found that SWD could expand their ideas and express their creativity through collaboration with their peers. Further, Nami & Asadnia (2024) adopting the multimodalities to create DST, she combines animated DS, Game-based DS, Social media DS, Synchronous collaborative DS and Micro DS to enhance incidental and intentional vocabulary learning. Fan (2024) asserted that DST can facilitate content knowledge, language proficiency and academic competence. In the context of medical-related majors, Fan explored DST to encourage students to develop content related to their context, composing a written text, making an oral script, and doing a voice over to their video presentation. The final result showed that students were more aware of rhetorical, linguistic and inter-semiotic choices.

Third, six studies focused on a virtual reality project employing *Spherical video-based virtual reality (SVVR), Immersive virtual reality (iVR), Virtual Interpreting Practice (VIP) app, Assemblr (AR), Virtual Business Professional (VBP), Google Tour Creator.* *Spherical video-based virtual reality* (SVVR) allows students to co-create content for travel books. Students possibly choose content and create a layout for their written work and then uploaded to YouTube, this kind of activity is empirically foster creativity and curiosity ( Sun Joo & Lee Jin, 2024). Similarly, *Immersive virtual reality* (iVR) was used to enhance students’ oral English skills and engagement. Shi et al. (2024) employed iVR to create VR videos by inputting the dialogue script, building the dialogue setting, sharing it through the VR platform, practicing and recording the dialogue into the VR system. Another study on VR involved *Virtual Interpreting Practice* (VIP) application to foster interpreting skills. Another system called *Assemblr* (AR) to design a three-fold pamphlet QR code contains 3D interactive content of university tours for freshmen. Lin & Wang (2023) indicated the effect of using AR was on learner’s perception of creativity and boosted their motivation towards learn with technology. Thereupon, *Virtual Business Professional* (VBP) was employed in the study of Angeles Mestre-Segarra & Ruiz-Garrido (2022) and empirically promoted language-content communication and fostered intercultural awareness through collaborative online international learning. The last system under review is *Google Tour Creator.* Lin & Wang (2021) assigned participants to create virtual tours platform to introduce their hometown to an international student. This study confirmed that the VR project could facilitate students’ efficacy in creative thinking, and that technical skills also benefit their English communication skills.

Fourth, the gaining attention in TEPBLL area is conducting video-making project. Five studies employed TEPBLL with the aid of *Google Docs, EverCam, PowerDirector, Movie Maker, YouTube,* and *Web 2.0* platform to their project. The collaboration between *Movie Maker* and *Google Docs* empirically enhanced students’ communicative performance by creating public service announcement (PSA) videos ( Greenblatt & McDonald, 2022). Nishioka (2016) conducted research on collaborative DST using a Web 2.0 based application. The results showed that learners strategically used their first language, grammatical terminology, and private speech in the collaborative knowledge construction process during the project. To promote intra-cultural understanding, Yeh & Fu (2023) research has been conducted on video creation using *Power Director* which implies the improvement of English communication and deepens cultural understanding.

Fifth, three studies on mobile instant messaging-based projects suggested *online discussion forum*, *WeChat*, and *Whatsapp* platforms. The study of Nami & Asadnia (2024) used a multimodal platform of *Whatsapp, slideshare, blogpage, inshot* and *Instagram* to implement word contextualization, and story presentation through a learner-generated digital story telling (DST) project. The results indicated that DST design, development, presentation, and evaluation positively enhanced learners’ perceptions towards DST-based vocabulary learning. Avci & Adiguzel (2017) further researched the use of *Whatsapp* to foster communication skills and found that mobile instant messaging had a positive effect on student performance. Yao (2022) conducted research on climate education using online discussion forums, which increased learners’ willingness to combat climate change. Sixth, the studies focus on the Video Conferencing project with the aid of *Machinima* ( Dooly & Sadler, 2016) and *Skype* ( Kato et al., 2023) platform, focusing on speaking skills. The last is an Audio-visual Translation project employs *SubESPSKills* and *Moodle.* Javier Avila-Cabrera & Corral Esteban (2021) proposed *SubESPSKills* innovative project on subtitling tasks in an English for Specific Purposes class to improve written production skills. The results indicated improvements in writing production skills and some language knowledge (e.g., words, structure, etc.). Furthermore, the language skill and knowledge along with student and teacher activities of TEPBLL also portrayed in Table 5.

**Table 5.** Language skills and technologies in TEPBLL.

Language skills and knowledge	Technologies	Student activities	Teacher activities
Speaking	Audio Visual Translation tool (AVT)	Listening task, writing essays, reading exercises and creative dubbing assignment.	Supervising and checking the project process
	iMovie	Students take part in 3-minute group video assignment that were associated to the learning context of their classroom textbook.	Offering assistance when necessary
	Immersive Virtual Reality (IVR)	Create VR video using presentation, practice, and production procedure.	Supervise and check the project process
	Virtual Interpreting Practice (VIP)	Students is given an interpreting task. Each task (source text/audio/video) is 2 until 8 minutes long or about 250–350 words/400–700 characters.	Teacher played the audio files of pre-recorded speeches for in-class practice and monitoring student activity
	Google Classroom	Content development, written text, multimodal elements, oral script and voice over narration, tech training, presentation.	Lead group discussion, guide students
	Skype	Dual nations students made pairs and communicate.	Monitoring
Listening	Audio Visual Translation tool (AVT)	Listening comprehension exercises, composing reverse subtitle for assigned video.	Not stated
Reading	Moodle	Reading exercise for reverse subtitling.	Explaining the use of Moodle
Writing	Wikipedia	Enhance an already-existing Wikipedia page or write a new one about course-related subject.	Assess Wikipedia articles published by students
	Audio Visual Translation tool (AVT)	Listening comprehension exercises, composing reverse subtitle for assigned video.	Organizing stages for project activities
	Google Tour Creator	Student created virtual tours using Google Tour Creator to showcase their hometown to a global audience.	Guide student to do the project
Vocabulary	WhatsApp/Telegram	Discussing through chat on designing multi-modal digital story, development, and presentation.	Reducing group conflict
Creative and Critical Thinking	Spherical video-based virtual reality (SVVR)	While using SVVR technology to produce online multimedia to complement their written work – which would then be posted on YouTube – groups were required to select content and design layout for their chapter first.	Make notes, recorded writing progress each group, provide feedback
	Google tour creator (VR)	Create multiple VR scenes and create a tour on the desktop with Google’s street-view technology.	Guide student to do the project
Intercultural Awareness	WeChat	Students were encouraged to use English to communicate their ideas about any topics based on their interest.	Make a group on WeChat and give comment
	PowerDirector	Create video introducing their local cultures.	Correcting student’s error
	Padlet and Facebook	Student-produced presentation slides and videos doing joint project related to SDGs.	Supervising the collaboration process
Content Knowledge	Canvas	Students collaborated to build a model of the water cycle and its potential relationship to air pollution. Second, students composed/write a response to the problem.	Established and presented the project and driving question were established

Overall, from the above results, it can be found that the learners had an overall positive attitude towards TEPBLL and their learning outcomes were also satisfactory. The review results in the same nuance support Dressler et al. (2020) and Casal & Bikowski (2020) findings about the benefit of project-based language learning using technology that improves language skills (speaking, listening, writing, reading, vocabulary acquisition), increase intercultural awareness, and triggers creative and critical thinking.

### Implications of TEPBLL

Based on the analysis the 31 articles, we classified and briefly summarized the articles from three dimensions: language acquisition, teacher’s roles, and potential research on TEPBLL. In the language acquisition dimension, the previously mentioned literature showed that task design and students’ technological pedagogical content knowledge had great impacts on TEPBLL. Teachers and coordinators in the language program perceived that the content being taught has implications for student motivation in learning language ( Nanni, 2021), higher levels of motivation and acquisition of relevant skills ( Konieczny & Eckert, 2022), and expanding learning experiences and effectiveness, making learning a joyful and pervasive process, improving learners’ autonomy, and critical thinking, and increasing their motivation and self-confidence ( Chan, 2022).

In the teacher roles dimension, the literature indicates that teachers are urged to inaugurate TEPBLL activities by describing technological knowledge and learning objectives to students. As for teacher facilitation, it is also recommended that the teacher should fully understand their students’ perception of language proficiency to set up content and language-related objectives ( Sun Joo & Lee Jin, 2024) the same nuance asserted by Jao et al. (2023) that the digital story-telling has its level, so that the teacher should assign the project by their level of language proficiency. Another recommendation is that teachers present practical tutorials and provide fruitful tips for the project. Moreover, teachers should assign specific roles and responsibilities to each group member to ensure everyone participates ( Huang, 2021), the teacher sets up staged project tasks and provides progressive feedback, since real-time corrective feedback sustains learner motivation.

Additionally, this review asserts five potential topics for further exploration of future research: (1) the exploration of potential technology used for TEPBLL that suites inclusive classrooms ( Boardman & Hovland, 2022); (2) possible influences of students’ emotional states on their project performance ( Shi et al., 2024); (3) facilitating content knowledge and language proficiency using robots ( Fan, 2024); (4) explore the challenges and limitations faced by students in the process of collaborative video-making to refine the process ( Yeh et al., 2020); and (5) a higher number of students may help to generalize the results ( Mohamadi, 2018).

## Discussion

The review results indicated that certain studies have integrated technology tools into PBLL and confirmed that creative student output is unattainable through conventional courses. The incorporation of technology into project-based language learning was the principal factor contributing to the efficacy of TEPBLL in enhancing language knowledge, as it heightened students’ engagement and intrinsic motivation, stimulated creative and critical thinking, fostered collaboration, refined essential communication skills, and exposed intercultural understanding, ultimately leading to successful learning outcomes ( Avci & Adiguzel, 2017; Garib, 2023; Greenblatt & McDonald, 2022; Hsieh et al., 2022; Nami & Asadnia, 2024). The results of our review indicated that the majority of learners expressed positive attitudes towards TEPBLL ( H.-W. Huang, 2021; Javier Avila-Cabrera & Corral Esteban, 2021; Lin & Wang, 2021). In this respect, TEPBLL is best understood not as a distinct instructional method, but as a pedagogical configuration in which technology, task design, and social interaction jointly shape learning processes.

Across the reviewed literature, technology (i.e., Virtual Reality) also regarded as a rejuvenating and relaxing learning experience that benefited students’ English skills, as the project was engaging and enabled autonomy. Although it is noteworthy that students with high creative self-efficacy tend to feel less pressure than those with low creative self-efficacy, the result of their EMI aspect (i.e. enjoyment, perceive competence, perceived choice, usefulness), yet the post-test score showed no difference ( Lin & Wang, 2021). In this way, technology-enhanced PBLL (i.e. immersive VR) consistently reported promotes social engagement by participating and interacting in the VR environment with their peers ( Shi et al., 2024).

It was also reported that TEPBLL could enrich subject-specific vocabulary ( Dugartsyrenova & Sardegna, 2019; Javier Avila-Cabrera & Corral Esteban, 2021; Tatzl, 2015), resulting in active engagement in communicative activities among students with high degree of autonomy and motivation ( Greenblatt & McDonald, 2022). Since PBLL offers a hands-on approach to a real work-life situations, virtual interpreting practice (VIP) allows students to experience and practice their interpreting competency in authentic and immersive interpreting practice. Moreover, students generally had a positive attitude towards VIP for interpreting learning ( Chan, 2022).

The reviewed studies indicate that technologies such as audio-visual translation (AVT), video-editing software (e.g., iMovie), immersive virtual reality, virtual interpreting practice (VIP) tools, and video conferencing platforms (e.g., Skype) have been frequently used within TEPBLL context and are often associated with reported improvements in learners’ speaking practice. As described in the literature, these technologies appear to facilitate opportunities for interaction, collaboration, and authentic communication by providing socially mediated and interactive learning environments (see Table 5).

Given the rapid evolution o f educational technologies, this review does not advocate for the adoption of specific tools. Instead, it suggests that future research should move beyond tool-centred comparisons towards analyses of technological affordances — such as interactivity, multimodality, and social presence — and how these affordances interact with pedagogical goals and learner characteristics ( Lin & Wang, 2021; Shi et al., 2024). Such an approach may offer a more sustainable analytical framework than focusing on individual technologies that may quickly become obsolete.

Importantly, the review also identifies contextual and pedagogical constrains that shape the outcomes of TEPBLL implementations. Mohamadi (2018), for instance, conducted a comparative study of PBL and e-PBL of English idiom knowledge and reported that students faced a challenge in using technology because of the quality of the Internet, which caused them to not enjoy the same benefits as the non-tech group. Similarly, Nishioka (2016) investigated a Web 2.0-based collaborative digital story-telling project but found that most proficient learners showed dissatisfaction in collaborating with less proficient learners, as it took a longer time to complete the task. However, Nishioka also suggested that language educators could boost the motivation and engagement of highly proficient learners by highlighting how collaborative learning with peers facilitates the acquisition of the target language within the project.

## Conclusion

Overall, this systematic review examined 31 SSCI-indexed empirical studies and found five conceptual frameworks in TEPBLL area including social constructivism theory, sociocultural theory, incidental vocabulary learning, communicative language learning and self-determination theory. Seven project types involving telecollaborative project, digital story-telling, virtual reality, video making, mobile instant messaging-based, video conferencing and audio-visual translation projects were promoted after the enhancement of TEPBLL in the classroom. Thus, eight language skills and knowledge would be improved after the application of TEPBLL: speaking skills, listening skills, reading skills, writing skills, vocabulary knowledge, creative and critical thinking skills, intercultural awareness, and content knowledge. We also discussed the recommendations of previous studies in detail and considered possible research topics for future research.

Thus, it is crucial to note that this review focused on identifying a limited number of empirical works that employed technology to support the process of language learning and that were published in SSCI; nevertheless, several significant publications on TEPBLL were not covered in this review. For example, Dressler et al. (2020) integrated content and language learning in project-based learning implies the concept of CLIL-infused TEPBLL, which supports vocational or content-related majors conducting PBLL. However, the review foci are the theories that were used in the empirical studies on TEPBLL or the technologies aimed at language enhancement, and the implications for future research. However, there are certain limitations of this review that might be considered in the following ways in future research: (1) The current study used a limited number of articles from a single resource, that is Web of Science, to maintain the quality of the review. Future research would benefit from drawing on a wider range of data sources, including non-SSCI journals, conference proceeding; as well as from. (2) Future studies may consider examining TEPBLL from diverse pedagogical and disciplinary perspectives.

## Ethics and consent

No ethics and consent were required.

## Data Availability

Dataset of Systematic Literature Review of Technology Enhanced Project-based Language Learning at https://doi.org/10.5281/zenodo.15792672 ( Fikria et al., 2025a) PRISMA Checklist for ‘A Systematic Review of Technology-Enhanced Project-Based Language Learning: Theoretical Frameworks, Project Types, and Implications for Future Research and Practice’. https://doi.org/10.5281/zenodo.15792956 ( Fikria et al., 2025c) Data associated with this article is provided in online repository under the terms of Creative Common Attribution 4.0 International (CC BY 4.0).
