# Supplementary material for: A Systematic Review of Technology-Enhanced Project-Based Language Learning: Theoretical Frameworks, Project Types, and Implications for Future Research and Practice
Source: F1000Res. 2025 Dec 27;14:775. Originally published 2025 Aug 8. [Version 2] doi: 10.12688/f1000research.165491.2 (PMC12811719; doi:10.12688/f1000research.165491.2)
Supplement: Peer review history

# Reviewer report: Reviewer response for version 2

**DOI:** 10.5256/f1000research.194301.r447127

**Published:** 2026-01-31

**Authors:** Jiawei Wang

**Affiliations:** 1 Universiti Sains Malaysia, George Town, Malaysia

The manuscript has reached an acceptable standard, but some parts still need improvement. Specific suggestions are as follows:

 1. The introduction is still somewhat lengthy, especially the paragraphs on pages 3–4 which contain overlapping information.

 2. The conclusion could be further enhanced in terms of theoretical depth. Two points should be emphasized:

 (1) TEPBLL, as a composite model of "task design + social interaction + multimodal technology," should have a clearer theoretical positioning.

 (2) Emphasize the potential of TEPBLL for cross-cultural collaborative research.

 3. Future research suggestions could include specific methodological aspects, such as a systematic comparison of the causal mechanisms of different PBLL task structures.

Are the rationale for, and objectives of, the Systematic Review clearly stated?

Yes

Is the statistical analysis and its interpretation appropriate?

Yes

If this is a Living Systematic Review, is the ‘living’ method appropriate and is the search schedule clearly defined and justified? (‘Living Systematic Review’ or a variation of this term should be included in the title.)

Not applicable

Are sufficient details of the methods and analysis provided to allow replication by others?

Yes

Are the conclusions drawn adequately supported by the results presented in the review?

Yes

Reviewer Expertise:

not applicable.

I confirm that I have read this submission and believe that I have an appropriate level of expertise to confirm that it is of an acceptable scientific standard.

# Reviewer report: Reviewer response for version 2

**DOI:** 10.5256/f1000research.194301.r445310

**Published:** 2026-01-16

**Authors:** Saifon Songsiengchai

**Affiliations:** 1 Rajamangala University of Technology, Krungthep, Thailand

1. This review is descriptive and narrative and not quantitative in nature. Statistical synthesis, or a meta-analysis, was not planned by this review due to wide variation in the included studies’ designs, measures of outcome, and reporting practice.

*For example, the reviewed studies employ different project designs, technologies, and assessment approaches, making direct statistical comparison inappropriate.*

*2. This* review is a one-time systematic summary with a constant search period, rather than a living systematic review. There is no provision for routine updating or a systematic search strategy. The purpose of the review is to offer an overview of technology-facilitated project-based language learning over time; thus, it also fails to meet the criterion for a living systematic review.

 For example, the review clearly defines a fixed search timeframe (2015–2024) rather than outlining procedures for continuous or periodic updates.

Are the rationale for, and objectives of, the Systematic Review clearly stated?

Yes

Is the statistical analysis and its interpretation appropriate?

Partly

If this is a Living Systematic Review, is the ‘living’ method appropriate and is the search schedule clearly defined and justified? (‘Living Systematic Review’ or a variation of this term should be included in the title.)

Partly

Are sufficient details of the methods and analysis provided to allow replication by others?

Yes

Are the conclusions drawn adequately supported by the results presented in the review?

Yes

Reviewer Expertise:

Teaching, Education, Coaching and Mentoring

I confirm that I have read this submission and believe that I have an appropriate level of expertise to confirm that it is of an acceptable scientific standard.

# Reviewer report: Reviewer response for version 1

**DOI:** 10.5256/f1000research.182132.r406178

**Published:** 2025-09-09

**Authors:** Ahmad Ridho Rojabi

**Affiliations:** 1 Universitas Islam Negeri Kiai Haji Achmad Siddiq Jember, East Java, East Java, Indonesia

1. This manuscript addresses the integration of technology and project-based learning in language teaching, which is highly relevant to current educational trends.

 2. The introduction does not properly highlight research gaps. The introduction emphasizes "reviewing what exists," but it's unclear why a systematic review of TEPBLL is necessary.

 For example, are previous studies scattered without synthesis? Has the theoretical framework not been drawn up? Has there been no comprehensive review of project types? These points should be highlighted.

 3. The method section adheres to PRISMA, increasing credibility as a systematic review. Database clarity and year range: We only used Web of Science (SSCI) from 2015 to 2024. The mention of four exclusion gates and three inclusion rules is significant for validity and transparency. Explained the coding process, which included reading, recording, grouping, and generating categories. Two researchers double-coded and discussed with a third researcher to improve reliability. However, there is some inconsistency in the terminology; for example, "techno collaborations" sounds strange; perhaps they mean technological collaborations.

 4. The conclusion indicates that this paper focuses on the theory, technology, and implications of TEPBLL. There is a section with recommendations for future research to guide the reader.

Are the rationale for, and objectives of, the Systematic Review clearly stated?

Yes

Is the statistical analysis and its interpretation appropriate?

Yes

If this is a Living Systematic Review, is the ‘living’ method appropriate and is the search schedule clearly defined and justified? (‘Living Systematic Review’ or a variation of this term should be included in the title.)

Yes

Are sufficient details of the methods and analysis provided to allow replication by others?

Yes

Are the conclusions drawn adequately supported by the results presented in the review?

Yes

Reviewer Expertise:

Technology-enhanced language learning, engagement, and social presence.

I confirm that I have read this submission and believe that I have an appropriate level of expertise to confirm that it is of an acceptable scientific standard.

# Response

**Published:** 2025-12-24

**Authors:** Ainun Fikria

**Affiliations:** Languages and Arts, Universitas Negeri Surabaya, Surabaya, East Java, Indonesia

We sincerely thank the reviewer for the constructive and thoughtful comments, as well as for recognizing the relevance and timeliness of the topic. The feedback has been very helpful in improving the clarity, rigor, and coherence of the manuscript. Our responses to each comment are provided below.

**Reviewer comment:**

*This manuscript addresses the integration of technology and project-based learning in language teaching, which is highly relevant to current educational trends.*

**Response:**

 We appreciate the reviewer’s positive assessment of the topic’s relevance. The increasing integration of digital technologies in project-based language learning contexts motivated us to undertake this systematic review, and we have maintained a strong focus on contemporary educational practices throughout the manuscript.

**Reviewer comment:**

*The introduction does not properly highlight research gaps… it is unclear why a systematic review of TEPBLL is necessary.*

**Response:**

 We agree with the reviewer that the research gap needed to be more explicitly articulated. In response, we have **revised the Introduction** to clearly highlight the necessity of this systematic review. The revised text now explicitly states that:

- Existing studies on technology-enhanced project-based language learning (TEPBLL) are **scattered across contexts, technologies, and educational settings**, with limited synthesis.
- Prior research has not systematically integrated **theoretical frameworks**, **technology-supported project types**, and **pedagogical implications** within a single review.
- No previous systematic review has comprehensively mapped how different project types and technologies are linked to language skills and learning outcomes.

These revisions clarify why a systematic review of TEPBLL is both timely and necessary, and they explicitly position the present study as addressing this gap.

**Reviewer comment:**

*The method section adheres to PRISMA… However, there is some inconsistency in the terminology; for example, “techno collaborations” sounds strange.*

**Response:**

 We thank the reviewer for acknowledging the methodological rigor of the study, including adherence to PRISMA guidelines, transparent database selection (Web of Science SSCI, 2015–2024), clear inclusion and exclusion criteria, and the double-coding procedure with a third reviewer to ensure reliability.

 Regarding terminology, we agree that clarity and consistency are essential. In the revised manuscript, we have reviewed the terminology used throughout the text and replaced ambiguous expressions such as *“techno collaborations”* with clearer and more standard terms, including **“technology-mediated collaboration”** or **“technology-supported collaboration,”** depending on context. This revision improves precision and readability without altering the intended meaning.

**Reviewer comment:**

*The conclusion indicates that this paper focuses on the theory, technology, and implications of TEPBLL, and includes recommendations for future research.*

**Response:**

 We appreciate this observation. In the revised version, the Conclusion has been further refined to ensure that it clearly synthesizes the key contributions of the review—namely, the theoretical foundations, technology-supported project types, and pedagogical implications of TEPBLL—without overlapping with the Results or Discussion sections. The recommendations for future research have been clarified and aligned with the identified gaps in theory use, methodological consistency, and reporting practices, thereby offering concrete guidance for subsequent studies.

 We are grateful for the reviewer’s insightful comments, which have helped us strengthen the articulation of the research gap, improve terminological clarity, and refine the overall structure of the manuscript. We believe that the revisions have significantly enhanced the clarity, rigor, and contribution of the study.

# Reviewer report: Reviewer response for version 1

**DOI:** 10.5256/f1000research.182132.r406177

**Published:** 2025-08-30

**Authors:** Muthmainnah Muthmainnah

**Affiliations:** 1 Teacher training and education faculty, Universitas Al Asyariah Mandar, Polewali, Sulawesi Barat, Indonesia

The paper addresses an important and timely topic with potential to make a meaningful contribution. However, significant revisions are required to improve methodological rigor, analytical depth, and clarity of conclusions.

 Major Points for Revision

1. Clarify the rationale and objectives, explicitly state the research gap this review addresses.
2. Strengthen methodological transparency,**** include detailed search strings, timeframe, PRISMA flow diagram, and reliability checks.
3. Enhance data analysis, where possible, provide effect sizes, correlations, or subgroup analyses; justify why meta-analysis was not attempted.
4. Moderate conclusions, ****ensure claims are proportionate to the evidence presented and highlight limitations clearly.
5. Improve structure and flow, some sections overlap (e.g., introduction and discussion) and could be streamlined for clarity.

Are the rationale for, and objectives of, the Systematic Review clearly stated?

Partly

Is the statistical analysis and its interpretation appropriate?

Partly

If this is a Living Systematic Review, is the ‘living’ method appropriate and is the search schedule clearly defined and justified? (‘Living Systematic Review’ or a variation of this term should be included in the title.)

Not applicable

Are sufficient details of the methods and analysis provided to allow replication by others?

Partly

Are the conclusions drawn adequately supported by the results presented in the review?

Partly

Reviewer Expertise:

English education, Education and  AIED

I confirm that I have read this submission and believe that I have an appropriate level of expertise to confirm that it is of an acceptable scientific standard, however I have significant reservations, as outlined above.

# Response

**Published:** 2025-12-24

**Authors:** Ainun Fikria

**Affiliations:** Languages and Arts, Universitas Negeri Surabaya, Surabaya, East Java, Indonesia

We thank the reviewer for the careful evaluation of our manuscript and for acknowledging the importance and timeliness of the topic. We appreciate the constructive feedback and have undertaken substantial revisions to strengthen methodological rigor, analytical depth, clarity of conclusions, and overall structure. Below, we respond to each major point in detail.

**Reviewer comment:**

*Clarify the rationale and objectives, explicitly state the research gap this review addresses.*

**Response:**

 We agree that a clearer articulation of the research gap is essential. In the revised manuscript, the **Introduction has been substantially rewritten** to explicitly state the need for this review. We now clearly argue that although technology-enhanced language learning and project-based learning have been widely studied, existing research on technology-enhanced project-based language learning (TEPBLL) remains **fragmented**, with no prior systematic review integrating **theoretical frameworks, technology-supported project types, targeted language skills, and pedagogical implications** within a single synthesis.

 The objectives of the review have been clarified and aligned with this gap through explicitly stated research questions that guide the scope and analytical focus of the study.

**Reviewer comment:**

*Strengthen methodological transparency, include detailed search strings, timeframe, PRISMA flow diagram, and reliability checks.*

**Response:**

 We have substantially strengthened the **Methods** section to improve transparency and reproducibility. The revised manuscript now includes:

- Explicit Boolean search strings used in the database search.
- Clear specification of the database (Web of Science SSCI) and review timeframe (2015–2024).
- A **PRISMA 2020 flow diagram** documenting identification, screening, eligibility, and inclusion stages.
- Detailed inclusion and exclusion criteria, including four exclusion gates and three inclusion rules.
- A clear description of the coding process and **interrater reliability analysis**, including double coding and calculation of Cohen’s kappa (κ = 0.808, 95% CI = 0.70–0.93), with disagreement resolution procedures.

These additions ensure that the review process is transparent, rigorous, and replicable.

**Reviewer comment:**

*Enhance data analysis, where possible, provide effect sizes, correlations, or subgroup analyses; justify why meta-analysis was not attempted.*

**Response:**

 We acknowledge the value of quantitative synthesis and have enhanced the analytical depth where feasible. The revised manuscript includes **descriptive subgroup analyses** based on theoretical frameworks, project types, technologies, and language skills.

 However, a meta-analysis was not conducted due to **substantial heterogeneity** in study designs, outcome measures, participant populations, and reporting practices. In addition, many primary studies did not report sufficient statistical information (e.g., effect sizes or variance estimates) to support valid quantitative aggregation. This rationale is now explicitly stated and justified in both the Methods and Discussion sections.

**Reviewer comment:**

*Moderate conclusions, ensure claims are proportionate to the evidence presented and highlight limitations clearly.*

**Response:**

 We have carefully revised the **Discussion and Conclusion** sections to moderate claims and avoid causal interpretations. Strong or definitive language has been replaced with cautious, evidence-aligned phrasing (e.g., “suggests,” “is associated with,” “indicates”).

 A dedicated discussion of **limitations** has been added, addressing database scope, heterogeneity of study designs, reliance on self-reported outcomes, and constraints in quantitative reporting. These revisions ensure that conclusions remain proportionate to the available evidence.

**Reviewer comment:**

*Improve structure and flow; some sections overlap and could be streamlined for clarity.*

**Response:**

 To address this concern, we have revised the manuscript structure to reduce redundancy and improve coherence. Conceptual framing has been consolidated in the Introduction, descriptive findings are confined to the Results, and interpretive analysis is clearly positioned in the Discussion. Transitions between sections have been refined to enhance readability and logical flow.

 We believe these revisions substantially strengthen the manuscript and directly address all major concerns raised by the reviewer. We are grateful for the insightful feedback, which has helped improve the clarity, rigor, and contribution of the study.
